# Prevalence and Determinants of Intestinal Parasitic Infections Among Diabetes Mellitus Patients at Debre Tabor Comprehensive Specialized Hospital, North‐Central Ethiopia: A Cross‐Sectional Study

**DOI:** 10.1002/hsr2.71406

**Published:** 2025-10-21

**Authors:** Shewaneh Damtie, Biruk Legese, Ayenew Berhan, Alemie Fentie, Birhanu Getie, Mulat Erkihun, Tegenaw Tiruneh, Teklehaimanot Kiros, Birhanemaskel Malkamu, Andargachew Almaw

**Affiliations:** ^1^ Department of Medical Laboratory Science College of Health Sciences Debre Tabor University Debre Tabor Ethiopia

**Keywords:** diabetes mellitus, immunocompromised, intestinal parasitic infections, prevalence, risk factors

## Abstract

**Background and Aims:**

Both intestinal parasitic infections and diabetes mellitus are major global health concerns, particularly in developing countries. The compromised immunity of diabetic patients increases their susceptibility to intestinal parasites. However, little is known about the burden of these infections among diabetic patients in Ethiopia, especially in the study area. Therefore, this study aimed to assess the prevalence and associated risk factors of intestinal parasitic infections among diabetic patients at Debre Tabor Comprehensive Specialized Hospital.

**Methods:**

A hospital‐based cross‐sectional study was conducted at Debre Tabor Comprehensive Specialized Hospital from December 1 to 30, 2024. A total of 262 participants were selected using systematic sampling. Data on sociodemographic and clinical factors were collected through a semi‐structured questionnaire. Stool samples were examined using direct wet mount, formol‐ether concentration, and modified Ziehl–Neelsen staining techniques. Data were entered into EPiData version 3.1 and analyzed using SPSS version 24. Logistic regression was employed to identify risk factors, with odds ratios and 95% confidence intervals calculated. A *p*‐value < 0.05 was considered statistically significant.

**Results:**

The overall prevalence of intestinal parasitic infection was 20.6% (95% CI: 15.9–26.0). Among the identified parasites, *Entamoeba histolytica/dispar* had the highest prevalence (26 cases, 9.9%), followed by *Cryptosporidium spp*. (15 cases, 5.7%) and *Giardia lamblia* (9 cases, 3.4%). Improper latrine utilization (AOR = 2.08, 95% CI: 1.13–3.81), consumption of unwashed vegetables or fruits (AOR = 3.62, 95% CI: 1.14–7.70), drinking well or spring water (AOR = 2.76, 95% CI: 1.45–5.27), and the presence of domestic animals in the house (AOR = 2.17, 95% CI: 1.18–3.98) were significantly associated with intestinal parasitic infections among diabetic patients.

**Conclusions:**

Intestinal parasitic infections are prevalent among diabetic patients, with key risk factors including improper latrine utilization, consumption of unwashed fruits or vegetables, drinking well or spring water, and the presence of domestic animals in the household.

**Abbreviations**
AOR: adjusted odds ratio
COR: crude odds ratio
DM: diabetes mellitus
IDF: International Diabetes Federation
IP: Intestinal Parasite
IPIs: intestinal parasite infection
OHAs: oral hypoglycemic agents

## 1 Introduction

Intestinal parasitic infections (IPIs) continue to pose a significant global health challenge, particularly in regions with inadequate sanitation [1, 2]. An estimated 3.5 billion people worldwide are at risk of various types of IPIs, with 450 million experiencing notable health impacts [3, 4]. Recent pooled data indicate that the prevalence of intestinal parasitic infections among diabetic patients in Africa is approximately 31% [5], highlighting an urgent need for preventive efforts in this high‐risk group. Intestinal parasitic infections remain a major public health concern, mostly in developing countries, due to the sanitation shortages, poor hygienic conditions, and neglect of health education [6, 7]. Ethiopia faces significant challenges with its drinking water quality and latrine coverage, which rank among the lowest globally. These issues, along with other risk factors, contribute to intestinal parasitic infections being the second leading cause of outpatient morbidity in the country [4].

Although most intestinal parasites (IPs) cause mild disease, some are recognized as opportunistic pathogens, capable of causing severe or life‐threatening complications in immunocompromised individuals [8]. The risk of developing an infection, the duration of the carrier state, the severity of complications, and the mortality rate associated with intestinal parasites are significantly elevated in individuals with immunosuppressive conditions such as acquired immunodeficiency syndrome (AIDS), cancer, or diabetes mellitus (DM) or those undergoing treatment with immunosuppressive drugs [9, 10].

DM is a medical condition characterized by chronic elevated blood glucose, caused by defects in insulin production, function, or both [11]. It may result from either the autoimmune destruction of insulin‐producing beta cells in the pancreas (type 1) or by insulin resistance (type 2), a condition in which the cells are unable to use insulin properly [12]. Worldwide, approximately 536.6 million adults between the ages of 20 and 79 are living with diabetes, and this figure is projected to reach 783.2 million by 2045 [13, 14]. In developing countries, the prevalence of diabetes mellitus is rising due to various factors, including urbanization, sedentary lifestyles, and changes in diet and exercise patterns [15]. The International Diabetes Federation (IDF) reports that in 2021, 24 million adults aged 20–79 in the IDF Africa Region were affected by diabetes, with a regional prevalence of 4.5%. This figure is expected to grow to 55 million by 2045 [16]. In Ethiopia, the prevalence of DM has risen from 3.8% to 5.2% [17].

Diabetic individuals are considered immunocompromised. Typically, both innate and adaptive immune responses are impaired in DM [4]. Diabetes affects the physiological and barrier functions of the intestine, weakens T‐cell function, and leads to alterations in humoral immunity [18]. Hyperglycemia diminishes the mobilization, chemotaxis, phagocytosis, and intracellular killing functions of polymorphonuclear leukocytes [19]. In addition, DM reduces the complement C4 component and various interleukins (ILs), including IL‐1, IL‐6, IL‐10, interferon‐gamma, and tumor necrosis factor [20]. Because of their overall weakened immune system, diabetic patients are more vulnerable to various opportunistic helminthic and protozoan infections [12].

Evaluating the burden of intestinal parasitic infections and identifying associated risk factors in diabetic patients is essential for improving their prognosis and overall health. In Ethiopia, only a limited number of studies have been published on the prevalence and associated factors of intestinal parasitic infections among DM patients [4, 12]. Moreover, the factors identified have not been reported consistently across studies. Additionally, no published research exists for the study area. Therefore, this study aimed to assess the prevalence and determinants of intestinal parasitic infections among diabetes mellitus patients at Debre Tabor Comprehensive Specialized Hospital, Northcentral Ethiopia.

## 2 Materials and Methods

### 2.1 Study Design, Period, and Settings

A hospital‐based cross‐sectional study was conducted at Debre Tabor Comprehensive Specialized Hospital from December 1 to 30, 2024. Debre Tabor is located in the northwest of the Amhara Region of Ethiopia, 103 km from Bahir Dar (the regional capital) and 666 km from Addis Ababa (the capital city of Ethiopia). The latitude and longitude of Debre Tabor are 11°50'18.6'' N and 38°05'58.3'' E, respectively. The hospital, one of the oldest in the Amhara Region, provides services to a population of approximately five million. It currently serves as a referral center for district hospitals in the zone and as a teaching hospital for medical and health science students. The hospital offers primary care for diabetes patients, including registering and treating all individuals diagnosed with diabetes.

### 2.2 Source and Study Population

The source population consisted of all diabetes patients who visited the chronic illness clinic at Debre Tabor Comprehensive Specialized Hospital during the study period. The study population included those patients who met the eligibility criteria.

### 2.3 Eligibility Criteria

Individuals with diabetes who were willing and able to provide written informed consent were included in the study. However, those with cancer, chronic diseases other than diabetes, or immunodeficiency disorders, as well as individuals currently receiving immunosuppressive medications or who had taken antibiotics or antiparasitic drugs within the 2 weeks preceding sample collection, were excluded. Additionally, pregnant women, critically ill individuals, those with severe mental disorders, or individuals with impairments in hearing and/or speech were also excluded from the study.

### 2.4 Sample Size Determination and Sampling Technique

The sample size was calculated using the single population proportion formula, considering a 19.2% prevalence of intestinal parasites reported in a previous similar study [4], a 95% confidence interval (*Z* = 1.96), and a 5% margin of error (*d* = 0.05). This yielded an initial sample size of 238. After accounting for a 10% nonresponse rate, the final sample size was adjusted to 262. A systematic sampling method was used to select participants, with the diabetic patients' record book as the sampling frame. With a total of 900 diabetic patients registered and attending the DM clinic, the sampling interval was calculated as *K* = 900/262 ≈ 3. The first patient record was selected by the lottery method, followed by the inclusion of every third record (*K* = 3) in the study.

### 2.5 Data Collection and Laboratory Methods

Data collection was conducted after obtaining informed consent from the participants. Information on sociodemographic characteristics, clinical factors, and other potential risk factors for intestinal parasitosis was gathered through face‐to‐face interviews using a pretested, semi‐structured questionnaire. Additionally, some clinical variables, such as the type of DM, duration of DM, and type of medication taken, were collected from the participants' medical records. Participants were classified as having type 1 or type 2 diabetes mellitus based on the diagnosis recorded in their medical records by the attending physician, following the hospital′s standard clinical diagnostic criteria.

Before stool sample collection, participants were given clear instructions on how to properly collect an adequate specimen. Approximately 5 g of fresh stool were obtained from each participant using a clean, dry, and leak‐proof container. Each sample container was accurately labeled with the participant′s name, case number, and the date and time of collection. The collected stool samples underwent both macroscopic and microscopic examination within 15 min of collection at Debre Tabor Comprehensive Specialized Hospital laboratory. Macroscopic analysis involved evaluating the stool′s color, consistency, and the presence or absence of mucus, blood, adult worms, or parasite body segments. Microscopic examination was carried out using direct saline and iodine wet mount techniques, along with the formal‐ether concentration method to detect intestinal parasites. Additionally, the modified Ziehl‐Neelsen staining technique was employed to identify oocysts of intestinal coccidia, such as *Cryptosporidium*, *Isospora*, and *Cyclospora* spp.

#### 2.5.1 Normal Saline and Iodine Preparations

A small amount of stool (1–2 mg) was mixed with 1–2 drops of normal saline (0.9%) or Lugol′s iodine solution. A coverslip was placed over the mixture, and the slide was examined under the 10× and 40× objectives of a light microscope. The saline wet mount technique was used to identify cysts and trophozoites of protozoa, as well as eggs or larvae of helminths. The iodine direct smear method enhanced the visualization of key protozoan features and facilitated differentiation between *Entamoeba histolytica/dispar* cysts and the commensal *Entamoeba coli* [21].

#### 2.5.2 Formol‐Ether Concentration Technique

This technique is widely used because it effectively concentrates a broad range of parasites while preserving their morphology, which is essential for detecting low parasite densities. One gram of each stool sample was mixed with 7 mL of 10% formalin solution in a centrifuge tube. The emulsified feces were filtered through a sieve into a test tube. Next, 3–4 mL of diethyl ether was added and mixed for 15 s. The resulting formol‐ether emulsion was centrifuged at 1500 rpm for 1 min. After centrifugation, the tube was inverted to decant the supernatant, leaving a few drops of sediment. The sediment was thoroughly mixed, and a drop was placed on a clean glass slide, covered with a coverslip, and examined under the 10× and 40× objectives of a light microscope [21].

#### 2.5.3 Modified Ziehl–Neelsen Method

A 15 mL conical centrifuge tube was filled with 1 g of fecal sample, 10 mL of saline, and a double layer of moist gauze for sieving. Then, 10 mL of 10% formalin and 3 mL of ether were added to the tube. The mixture was centrifuged at 2000 rpm for 5 min. The concentrated stool sediment was used to prepare a thin smear, which was air‐dried before staining using the modified Ziehl–Neelsen method to detect oocysts.

The air‐dried smear was stained with unheated carbol fuchsin for 15 min and then rinsed with water. After rinsing, 1% acid alcohol was applied for 15 s to decolorize the smear, followed by another rinse with water. A counterstain of 0.5% methylene blue was applied for 30 s. The smear was rinsed, allowed to air dry, and examined under a light microscope at low‐power magnification and using the oil immersion objective to detect and identify oocysts [22].

### 2.6 Data Quality Assurance

Before the data collection began, the questionnaire was pretested for accuracy and consistency on 5% of the sample size at Addis Zemen Primary Hospital, located outside the study area. A 1‐day training session was conducted for the data collectors, covering essential topics such as the study's objectives and significance, confidentiality, informed consent, participants' rights, interviewing techniques, sample collection, laboratory procedures, and quality control practices. To ensure the accuracy and consistency of the collected data, the investigators closely supervised the data collection process daily, providing continuous feedback to the data collectors. The completeness, accuracy, and clarity of the gathered data were meticulously reviewed to maintain high‐quality results.

For each stool sample, two slides were prepared and examined by two different laboratory technologists. The quality of laboratory test results was rigorously ensured by strictly following the standard operating procedures and laboratory manuals of Debre Tabor Comprehensive Specialized Hospital Laboratory, from the pre‐analytical phase of sample collection to the post‐analytical phase of result interpretation. All laboratory tests were conducted only after the completion of the necessary quality control procedures, ensuring the safety and reliability of the methods used.

### 2.7 Data Analysis and Interpretation

To ensure accuracy, the data were cleaned, validated, and thoroughly reviewed. After being entered into EPI Data 3.1, the data were transferred to SPSS version 24 for statistical analysis. Descriptive statistics, including frequency distributions, summary measures, and variability metrics, were computed. Bivariate and multivariate logistic regression analyses were performed to examine the relationships between independent and dependent variables. To minimize the impact of confounding factors, variables with a *p*‐value of < 0.25 in the bivariate analysis were included in the multivariable model. All statistical tests were two‐sided, and a *p*‐value of less than 0.05 was considered statistically significant.

### 2.8 Ethical Considerations

Ethical approval was obtained from the Research and Ethical Review Committee of Debre Tabor University (reference number: CHS/122/2024). Permission letters were also obtained from the medical director of Debre Tabor Comprehensive Specialized Hospital and the head of the DM clinic. The study adhered to the principles of the Helsinki Declaration. Written informed consent was obtained from all participants. For illiterate participants, consent was provided by their legal guardians, while for those under 18 years of age, consent was obtained from parents or legal guardians. No financial compensation or other incentives were offered to participants. To protect confidentiality, codes were assigned to participants, and access to the collected data was restricted to authorized personnel only. Participants diagnosed with intestinal parasitic infections were referred to the appropriate department at Debre Tabor Comprehensive Specialized Hospital for treatment.

## 3 Results

### 3.1 Socio‐Demographic Characteristics of Participants

This study included a total of 262 patients with diabetes mellitus. The participants had a mean age of 39.1 ± 12.4 years, with 38.5% being over 45 years old. Males accounted for more than half of the participants (52.7%). The majority (65.9%) resided in urban areas. Most participants (91.6%) identified as followers of the Orthodox religion. Furthermore, 71.8% were married, and 31.7% had no formal education (Table 1).

**Table 1:** Socio‐demographic characteristics of diabetic patients attending Debre Tabor Comprehensive Specialized Hospital, North‐central Ethiopia, 2024 (*N* = 262).

Variables	Category	Frequency	Percentage
Age (years)	< 15	26	9.9
	15–30	45	17.2
	31–45	90	34.4
	> 45	101	38.5
Sex	Male	138	52.7
	Female	124	47.3
Residence	Urban	170	64.9
	Rural	92	35.1
Religion	Orthodox	240	91.6
	Muslim	17	6.5
	Protestant	5	1.9
Marital status	Never married	48	18.3
	Married	188	71.8
	Widowed	10	3.8
	Divorced/separated	16	6.1
Educational status	No formal education	83	31.7
	Primary school	65	24.8
	High school	44	16.8
	College/University	70	26.7
Occupation	Government employed	53	20.2
	Private employed	32	12.2
	Housewife	56	21.4
	Merchant	48	18.3
	Farmer	36	13.7
	Student	22	8.4
	Daily laborer	15	5.7

### 3.2 Prevalence of Intestinal Parasites

**Figure 1:**
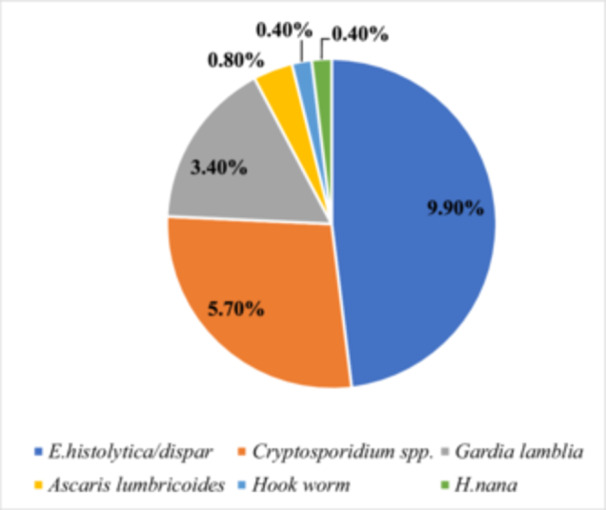
Frequency of intestinal parasites among diabetic patients attending Debre Tabor Comprehensive Specialized Hospital, Northwest Ethiopia, 2024 (*N* = 262).

Out of 262 study participants, intestinal parasites were identified in 54 diabetic patients (20.6%, 95% CI: 15.9–26.0), whereas 208 (79.4%) tested negative for IP. A total of six different species of intestinal parasites were identified, comprising three protozoan species and three helminth species. Among the detected parasites, *E. histolytica*/*dispar* had the highest prevalence (26 cases, 9.9%), followed by *Cryptosporidium spp*. (15 cases, 5.7%) and *Giardia lamblia* (9 cases, 3.4%) (Figure 1). The majority of infected diabetic patients had a single infection (96.3%), while 3.7% had a dual infection involving *E. histolytica/dispar* and *G. lamblia*.

### 3.3 Factors Associated With Intestinal Parasitic Infection Among Diabetic Patients

Rural residence, educational status, handwashing habits after defecation, latrine utilization, consumption of unwashed vegetables or fruits, source of drinking water, and the presence of domestic animals at home were all found to be associated with intestinal parasitic infection in a bivariate logistic regression analysis. However, in the multivariable logistic regression analysis, improper latrine utilization, consumption of unwashed vegetables/fruits, use of well or spring water for drinking, and the presence of domestic animals at home were independently associated with intestinal parasitic infection.

Consequently, diabetic patients who did not use a latrine had a 2.08 times higher likelihood of being infected with intestinal parasites compared to those who did (adjusted odds ratio [AOR] = 2.08, 95% CI: 1.13–3.81). Similarly, participants who consumed unwashed vegetables/fruits had 3.62 times higher odds of infection compared to their counterparts (AOR = 3.62, 95% CI: 1.14–7.70). Additionally, those who relied on well or spring water for drinking were 2.76 times more likely to be infected with intestinal parasites than individuals who used tap water (AOR = 2.76, 95% CI: 1.45–5.27). Moreover, DM patients who had domestic animals at home were 2.17 times more likely to contract intestinal parasites than those who did not (AOR = 2.17, 95% CI: 1.18–3.98) (Table 2).

**Table 2:** Bivariate and multivariable analysis of factors associated with intestinal parasitic infections among diabetic patients attending Debre Tabor Comprehensive Specialized Hospital, North‐central Ethiopia, 2024 (*N* = 262).

Variables	Category	IPI		COR (95% CI)	*p*‐value	AOR (95% CI)	*p*‐value
		Positive	Negative				
Age	< 15	6 (23.1)	20 (76.9)	1			
	15–30	8 (17.8)	37 (82.2)	0.72 (0.22–2.37)	0.59		
	31–45	19 (21.1)	71 ((78.9)	0.89 (0.31–2.53)	0.83		
	> 45	21 (20.8)	80 (79.2)	0.88 (0.31–2.45)	0.79		
Sex	Male	32 (23.2)	106 (76.8)	1.39 (0.76–2.57)	0.28		
	Female	22 (17.7)	102 (82.3)	1			
Residence	Urban	28 (16.5)	142 (83.5)	1		1	
	Rural	26 (28.3)	66 (71.7)	1.99 (1.09–3.67)	0.03	2.16 (0.98–4.75)	0.07
Educational status	No formal education	22 (26.5)	61 (73.5)	2.44 (1.04–5.74)	0.04	1.78 (0.73–4.34)	0.12
	Primary school	13 (20)	52 (80)	1.69 (0.67–4.28)	0.26	1.49 (0.57–3.83)	0.22
	High school	10 (22.7)	34 (77.3)	1.99 (0.74–5.38)	0.17	1.62 (0.59–4.07)	0.14
	College/University	9 (12.9)	61 (87.1)	1		1	
Swimming habit	Yes	9 (20)	36 (80)	0.96 (0.43–2.13)	0.91		
	No	45 (20.7)	172 (79.3)	1			
Washing hands before meals	Yes	48 (19.8)	195 (80.2)	1		1	
	No	6 (31.6)	13 (68.4)	1.88 (0.68–5.19)	0.23	1.58 (0.53–4.67)	0.32
Habit of Hand washing after defecation	Yes	38 (17.8)	175 (82.2)	1		1	
	No	16 (32.7)	33 (67.3)	2.23 (1.12–4.46)	0.02	2.32 (0.96–5.02)	0.06
Shoe wearing habit	Yes	43 (21.5)	157 (78.5)	1			
	No	11 (17.7)	51 (82.3)	0.79 (0.39–1.64)	0.52		
Latrine utilization	Yes	26 (15.8)	139 (84.2)	1		1	
	No	28 (28.9)	69 (71.1)	2.17 (1.18–3.98)	0.01	2.08 (1.13–3.81)	**0.03**
Eating unwashed vegetables/fruits	Yes	35 (30.4)	80 (69.6)	2.94 (1.58–5.50)	< 0.001	3.62 (1.14–7.70)	**0.001**
	No	19 (12.9)	128 (87.1)	1		1	
Habit of eating raw meat	Yes	16 (17.6)	75 (82.4)	0.75 (0.39–1.43)	0.38		
	No	38 (22.2)	133 (77.8)	1			
Source of drinking water	Well or spring water	28 (31.8)	60 (68.2)	2.66 (1.44–4.90)	0.002	2.76 (1.45–5.27)	**0.007**
	Tap water	26 (14.9)	148 (85.1)	1		1	
Domestic animal at home	Yes	29 (29.6)	69 (70.4)	2.34 (1.27–4.29)	0.006	2.17 (1.18–3.98)	**0.01**
	No	25 (15.2)	139 (84.8)	1		1	
Type of DM	Type 1	20 (22.2)	70 (77.8)	1.56 (0.62–2.16)	0.64		
	Type 2	34 (19.8)	138 (80.2)	1			
Duration of DM	< 5 year	25 (19.4)	104 (80.6)	1			
	5–10 year	19 (19.8)	77 (80.2)	1.03 (0.53–1.99)	0.94		
	> 10 year	10 (27.0)	27 (73.0)	1.54 (0.66–3.59)	0.32		
Type of antidiabetic drug	OHAs	25 (18.9)	107 (81.1)	1			
	Insulin	23 (21.1)	86 (78.9)	1.14 (0.61–2.16)	0.68		
	OHAs and Insulin	6 (28.6)	15 (71.4)	1.71 (0.60–4.85)	0.31		

*Note:* < 0.05 (bolded) indicate a statistically significant association.

## 4 Discussion

Intestinal parasites pose a major public health threat, particularly to immunocompromised individuals, as they can cause serious infections. These pathogens primarily affect those with weakened cellular immunity, increasing their susceptibility to opportunistic infections. In recent years, emerging intestinal parasites have received significant attention due to their involvement in clinically relevant infections among high‐risk populations. Diabetes mellitus is associated with immunosuppression, which may impair the body′s ability to eliminate intestinal parasites effectively [23]. Therefore, evaluating the prevalence of intestinal parasitic infections and identifying related risk factors in diabetic patients is essential for improving their prognosis and overall health.

The current prevalence of intestinal parasitic infection among diabetic patients was 20.6% (95% CI: 15.9–26.0). This result aligns with studies from Gondar, Ethiopia (19.2%) [4]; Arba Minch, Southern Ethiopia (19.5%) [12]; Nigeria (21.9%) [24]; and Turkey (16.9%) [25]. The similarities may be due to the use of comparable laboratory techniques for detecting intestinal parasites, along with similar populations and sample sizes. However, our finding was lower than those reported in Yemen (38.6%) [26], Egypt (44%) [10], Libya (40%) [27], India (36.6%) [28], Iraq (36.8%) [29], and the pooled prevalence of intestinal parasite infection among DM patients in Africa (31%) [5]. Conversely, our prevalence was higher than studies conducted in Ghana (12.5%) [30], Cameroon (10%) [9], Iran (12.6%) [31], and Chhattisgarh, India (14.4%) [32]. The differences observed may be attributed to various factors, including variations in the study populations (our study included both type 1 and type 2 diabetes patients, whereas some studies included only type 2 diabetes; our study also included all age groups, while other studies included only adults), study period, sample sizes, laboratory techniques used to detect intestinal parasites (e.g., the Egypt study used modified Trichrome staining and culture), personal hygiene practices, environmental sanitation, health promotion efforts, climatic conditions, geographic locations, and socioeconomic differences among the populations.

The most frequently detected intestinal parasite among diabetic patients in our study was *E. histolytica/dispar* (9.9%), which is consistent with findings from Yemen, Cameroon, Iraq, and Karnataka, India, where *E. histolytica/dispar* was also the predominant parasite among diabetic patients [9, 26, 28, 29]. Conversely, studies conducted in Gondar, Ethiopia; Arba Minch (Southern Ethiopia); Egypt; Libya; Nigeria; and Ghana reported *Ascaris lumbricoides*, *Cryptosporidium spp*., *Blastocystis*, *E. coli*, and *G. lamblia* as the more prevalent intestinal parasites, respectively [4, 10, 12, 24, 27, 30]. These differences may be attributed to variations in environmental conditions, geographic characteristics, levels of endemicity, and behavioral factors that influence parasite transmission and epidemiological patterns. A major limitation of this study is the inability to differentiate between morphologically indistinguishable *Entamoeba* species using molecular techniques. Traditional microscopy cannot distinguish between *E. histolytica*, which is pathogenic, and nonpathogenic species such as *E. dispar*. Consequently, the prevalence of pathogenic *E. histolytica* may be overestimated. Future studies employing molecular or antigen detection methods are warranted to provide more accurate species‐level identification.

This study examined multiple potential risk factors associated with intestinal parasitic infections. The findings revealed that IPIs were significantly linked to improper latrine utilization, consumption of unwashed vegetables or fruits, use of drinking water from wells or springs, and the presence of domestic animals in the household. Diabetic patients who did not use a latrine were 2.08 times more likely to be infected with intestinal parasites than those who did (AOR = 2.08, 95% CI: 1.13–3.81). This result is consistent with a previous study conducted in Gondar, Ethiopia [4]. The study emphasizes that inadequate latrine use and open defecation contribute to environmental and water contamination, which play a crucial role in the transmission of intestinal parasites.

The current study identified a link between the consumption of unwashed vegetables or fruits and intestinal parasite infection. Participants who regularly consumed unwashed vegetables or fruits had 3.62 times higher odds of contracting intestinal parasites compared to those who did not (AOR = 3.62, 95% CI: 1.14–7.70). This finding aligns with a study conducted in Yemen [26]. Unwashed fruits and vegetables may carry parasite eggs, cysts, or larvae due to contact with contaminated soil, water, fecal matter, or improper handling during cultivation, transportation, and storage. Therefore, it is essential to educate diabetic patients on the importance of food and hand hygiene in preventing and managing intestinal parasitic infections.

Drinking well or spring water was another notable risk factor identified in this study. Individuals who depended on well or spring water for drinking were 2.76 times more likely to contract intestinal parasites compared to those who used tap water (AOR = 2.76, 95% CI: 1.45–5.27). This finding aligns with findings of a previous study [33]. The higher risk is linked to the fecal‐oral transmission of these parasites as well, or spring water is more vulnerable to contamination than tap water. Unlike tap water, these sources are more prone to contamination from human and animal feces, particularly in areas lacking proper sanitation. Parasitic cysts and eggs can infiltrate these water sources through runoff from contaminated soil, agricultural practices, or open defecation.

The present study found an association between the presence of domestic animals in the household and intestinal parasite infection among diabetic patients. Diabetic patients who had domestic animals at home were 2.17 times more likely to contract intestinal parasites than those who did not (AOR = 2.17, 95% CI: 1.18–3.98). This finding is similar to a study conducted in Southern Ethiopia [12]. *Cryptosporidium spp*. and *G. lamblia*, which were the second and third most prevalent parasites in our study, respectively, can be transmitted to humans from animals through both direct and indirect contact [34, 35, 36]. Humans can ingest infective cysts or oocysts from contaminated food, water, or surfaces that have been exposed to animal feces. Close contact between humans and domestic animals in households, especially in areas with limited sanitation, increases the risk of zoonotic transmission.

### 4.1 Limitations of the Study

Firstly, this was a cross‐sectional study and, therefore, it was not possible to establish a causal relationship between intestinal parasite infection and the identified risk factors. Secondly, due to limited resources, we were unable to apply molecular techniques such as PCR to distinguish between pathogenic *E. histolytica* and *E. dispar*, which may have resulted in an overdiagnosis of amoebiasis. Thirdly, the study did not assess the intensity of infections for the different parasitic species. Lastly, since our research was conducted at a single hospital, the findings may not be fully generalizable to all diabetes patients in other regions or healthcare settings.

## 5 Conclusion

The current study revealed a high prevalence of intestinal parasitic infections among diabetic patients. Among the detected intestinal parasites, *E. histolytica/dispar* had the highest prevalence, followed by *Cryptosporidium spp*., and *G. lamblia*. Among the various potential risk factors assessed in this study, improper latrine utilization, consumption of unwashed fruits or vegetables, drinking water from wells or springs, and the presence of domestic animals in the house were significant risk factors associated with intestinal parasitic infection among diabetic patients.

Regular screening and treatment of intestinal parasitic infections are vital for maintaining the health of individuals with diabetes mellitus. Stakeholders should intensify efforts to raise awareness about infection control, improve hygiene practices, ensure access to clean water, and promote the construction of public sanitation facilities. The current deworming program in Ethiopia primarily focuses on children and pregnant women. Therefore, we recommend expanding the national deworming program to include adults with chronic conditions such as diabetes mellitus, who constitute an overlooked high‐risk group. Routine screening for intestinal parasitic infections and periodic deworming interventions should be integrated into diabetes care to reduce infection rates and prevent related complications. Additionally, incorporating targeted health education on hygiene practices and safe water use into diabetes management programs could strengthen existing control efforts and improve patient outcomes.

## Author Contributions

**Shewaneh Damtie:** conceptualization, investigation, writing – original draft, methodology, visualization, writing – review and editing, software, formal analysis, data curation, project administration, resources, supervision. **Biruk Legese:** conceptualization, investigation, validation. **Ayenew Berhan:** validation, writing – review and editing, conceptualization, resources. **Alemie Fentie:** conceptualization, investigation, writing – original draft, visualization. **Birhanu Getie:** conceptualization, validation, data curation. **Mulat Erkihun:** investigation, visualization, software. **Tegenaw Tiruneh:** writing – review and editing; formal analysis. **Teklehaimanot Kiros:** investigation, supervision. **Birhanemaskel Malkamu:** conceptualization, visualization, supervision. **Andargachew Almaw:** conceptualization, writing – review and editing, supervision, formal analysis, methodology, project administration.

## Conflicts of Interest

The authors declare no conflicts of interest.

## Transparency Statement

The lead author Shewaneh Damtie affirms that this manuscript is an honest, accurate, and transparent account of the study being reported; that no important aspects of the study have been omitted; and that any discrepancies from the study as planned (and, if relevant, registered) have been explained.

## Data Availability

The authors confirm that the data supporting the findings of this study are available in the article.
